# *Chlamydia trachomatis, Neisseria gonorrhoea*, and *Trichomonas vaginalis* infections among pregnant women and male partners in Dutch midwifery practices: prevalence, risk factors, and perinatal outcomes

**DOI:** 10.1186/s12978-021-01179-8

**Published:** 2021-06-26

**Authors:** Eline L. M. Op de Coul, Demi Peek, Yolanda W. M. van Weert, Servaas A. Morré, Ingrid Rours, Chantal Hukkelhoven, Ank de Jonge, Birgit van Benthem, Monique Pereboom

**Affiliations:** 1 Centre for Infectious Disease Control, National Institute for Public Health and the Environment (RIVM), P.O. Box 1, 3720 BA Bilthoven, The Netherlands; 2 Laboratory of Immunogenetics, Department of Medical Microbiology and Infection Control, VU University Medical Center, Amsterdam, The Netherlands; 3 Institute for Public Health Genomics (IPHG), Department of Genetics and Cell Biology, Research School GROW (School for Oncology and Developmental Biology), Faculty of Health, Medicine and Life Sciences, University of Maastricht, Maastricht, The Netherlands; 4 Kinderplein, Medical Centre for Quality of Life, Rotterdam, The Netherlands; 5 Perined, Utrecht, The Netherlands; 6 Amsterdam UMC, Vrije Universiteit Amsterdam, Midwifery Science, AVAG, Amsterdam Public Health, Amsterdam, The Netherlands

**Keywords:** Chlamydia, Gonorrhoea, Trichomonas, STI, Pregnancy, Midwifery practice, Adverse perinatal outcome, Prematurity

## Abstract

**Background:**

Antenatal screening for HIV, syphilis and HBV has been successfully implemented in The Netherlands, but data on other STI among pregnant women or male partners are limited. Our objectives: (i) to assess the prevalence of *Chlamydia trachomatis* (CT), *Neisseria gonorrhoeae* (NG) and *Trichomonas vaginalis* (TV) among pregnant women and male partners, (ii) to identify risk factors for these STI during pregnancy, and (iii) to identify adverse perinatal outcomes (APO) associated with STI.

**Methods:**

Cross-sectional study. Pregnant women aged ≤ 30 years (n = 548) and male partners (n = 425) were included at 30 midwifery practices during 2012–2016. Participants provided a self-collected vaginal swab (women) or urine sample (men) and completed a questionnaire. Perinatal data were derived from pregnancy cards. APO was defined as premature rupture of membranes, preterm delivery, low birthweight, stillbirth, neonatal conjunctival and respiratory infections. Data were analysed by logistic regression.

**Results:**

STI were present in 2.4% of pregnant women (CT 1.8%, NG 0.4%, TV 0.4%), and in 2.2% of male partners (CT 2.2%, NG 0.2%, TV 0%). Of young women (≤ 20 years), 12.5% had a CT infection. Prevalent STI during pregnancy was associated with female young age (≤ 20 years vs ≥ 21 years) (adjusted OR 6.52, CI 95%: 1.11–38.33), male non-Western vs Western background (aOR 9.34, CI 2.34–37.21), and female with ≥ 2 sex partners < 12 months vs 0–1 (aOR 9.88, CI 2.08–46.91). APO was not associated with STI, but was associated with female low education (aOR 3.36, CI 1.12–10.09), complications with previous newborn (aOR 10.49, CI 3.21–34.25 vs no complications) and short duration (0–4 years) of relationship (aOR 2.75, CI 1.41–5.39 vs ≥ 5 years). Small-for-gestational-age was not associated with STI, but was associated with female low education (aOR 7.81, 2.01–30.27), female non-Western background (aOR 4.41, 1.74–11.17), and both parents smoking during pregnancy (aOR 2.94, 1.01–8.84 vs both non-smoking).

**Conclusions:**

Prevalence of STI was low among pregnant women and male partners in midwifery practices, except for CT among young women. The study could not confirm previously observed associations between STI and APO, which is probably due to low prevalence of STI, small study sample, and presumed treatment for STI.

**Supplementary Information:**

The online version contains supplementary material available at 10.1186/s12978-021-01179-8.

## Plain language summary

Antenatal screening for HIV, syphilis and HBV has been successfully implemented in The Netherlands, but data on other STI among pregnant women or male partners are limited. Our objectives were: (i) to assess the prevalence of *Chlamydia trachomatis* (CT), *Neisseria gonorrhoeae* (NG) and *Trichomonas vaginalis* (TV) among pregnant women and male partners, (ii) to identify risk factors for these STI during pregnancy, and (iii) to identify adverse perinatal outcomes (APO) associated with STI.

Pregnant women aged ≤ 30 years and male partners were included at 30 midwifery practices. Women provided a vaginal swab, partners a urine sample; both completed a questionnaire. Perinatal data were derived from midwives.

STI were present in 2.4% of pregnant women (CT 1.8%, NG 0.4%, TV 0.4%), and in 2.2% of male partners (CT 2.2%, NG 0.2%, TV 0%). Of women ≤ 20 years, 12.5% had a CT infection. Prevalent STI during pregnancy was associated with female young age, male non-Western background, and female with ≥ 2 sex partners < 12 months. APO was not associated with STI, but was associated with female low education, complications with previous newborn, and short duration of the relationship. Small-for-gestational-age was not associated with STI, but was associated with female low education, female non-Western background, and both parents smoking during pregnancy.

Prevalence of STI was low among pregnant women and male partners in midwifery practices, except for CT among young women. The study could not confirm previously observed associations between STI and APO. Probably due to low prevalence of STI, small study sample, and presumed treatment for STI.

**Supplementary Information:**

The online version contains supplementary material available at 10.1186/s12978-021-01179-8.

## Background

In the Netherlands, pregnant women are screened for HIV, syphilis and hepatitis B [1, 2], but not for other sexually transmitted infections (STI). Hence, limited data are available on STI such as *Chlamydia trachomatis* (CT), *Neisseria gonorrhoeae* (NG) or *Trichomonas vaginalis* (TV) among pregnant women and their male partners. The World Health Organization (WHO) estimated for 2012 that among women of reproductive age (15–49 years) in the European region the prevalence of CT was 2.2% (95% uncertainty interval (UI): 1.6–2.9%), NG was 0.3% (0.2–0.5%), and TV was 1.0% (0.8–1.3%). For men, these prevalence rates were 1.5% (0.9–2.6%), 0.3% (0.2–0.5%), and 0.1% (0.1–0.2%) respectively [3].

An accurate detection and effective management of these STI are particularly relevant to women in their reproductive age as most infections are asymptomatic and may affect reproductive health [4, 5]. Untreated infections with CT, NG or TV in women can result in pelvic inflammatory disease (PID), which can cause chronic pelvic pain, infertility, and ectopic pregnancies [6–8]. During pregnancy, these STI have been associated with adverse perinatal outcomes (APO) including premature rupture of membranes (PROM), preterm delivery, low birthweight [9–19], stillbirth, and neonatal conjunctival and respiratory infections [20–23]. The effect of these STI on newborns who are born small for gestational age (SGA) is less evident [23, 24].

Although having STI during pregnancy is considered a risk factor for APO [15, 16, 23], data regarding STI prevalence rates in pregnant women together with their male partners, and factors associated with STI in pregnancy or its associations with APO are limited when taking into account sociodemographic and lifestyle factors [12, 25–28]. Also, findings from observational and cohort studies are inconclusive about the strengths of the associations between STI and reproductive outcomes [23, 24].

In many countries CT, NG and TV infections in pregnant women are still identified and treated by syndromic management [29]. Antenatal screening, especially for NG and TV, is often limited to resource-limited regions [30–33], where prevalence rates and incidence rates are much higher compared to high-income regions [29, 34, 35]. Although international guidelines recommend antenatal screening for CT of all women under 25 years of age and of older women at increased (sexual) risk [7], in the Netherlands these recommendations are not followed.

A previous study among pregnant women in Rotterdam showed an overall CT prevalence of 3.9% (including women up to ≥ 30 years of age). Women who tested positive for CT had higher adjusted odds ratios (aORs) for preterm delivery at 32 weeks (aOR 4.3, 95%CI 1.3–3.5) and 35 weeks (aOR 2.7, 95% CI 1.1–6.5) of pregnancy compared to CT negative women [36]. For the Netherlands CT screening of all pregnant women is considered cost-saving [37], based on the prevalence of 3.9% from the Rotterdam study [36]. Cost-effectiveness studies on screening for NG and TV infections among pregnant women in the Netherlands or other high-income regions are greatly lacking, and more insight is needed in these prevalence rates among pregnant women and their partners.

To inform recommendations about antenatal screening for these STI, we conducted a national cross-sectional study with the following aims: (i) to assess the CT-, NG-, and TV prevalence rates among pregnant women aged ≤ 30 years and their male partners who present to midwifery practices, (ii) to explore risk factors associated with these STI during pregnancy, and (iii) to explore the association between STI during pregnancy and adverse perinatal outcomes, with special focus on CT infection*.*

## Methods

### Study participants

The design of this multicentre observational study that included thirty midwifery practices across the Netherlands is described in detail elsewhere [38, 39]. The practices included covered approximately 5.0% of the pregnant women in the Netherlands (Supplementary file). In brief, pregnant women between 18 and 30 years of age were enrolled by their midwives if they could understand Dutch. There were no restrictions to the gestational age at inclusion. Male partners were included without an age limit, if they were present at the time of their partners’ enrolment and could understand Dutch. Both, women and partners signed an Informed Consent (IC) form for participation including follow-up data collection on perinatal outcomes through their midwives (pregnancy cards). Subsequently, they received a test package including home sampling devices, an information flyer, and a questionnaire for the woman and partner.

### Sample and data collection

Pregnant women filled in a questionnaire including sociodemographic characteristics (age, migration background, education, religion, marital status, postal code), obstetric characteristics (duration of pregnancy, parity, gravity, complaints, early and late miscarriages, abortions, complications with last pregnancy or previous newborn including delivery at ≤ 37 weeks, premature contractions, PROM, birthweight ≤ 2500 g, previous newborn with pneumonia or eye inflammation), biometric measures (weight, length, body mass index (BMI)), lifestyle factors (smoking, drug and alcohol use), antibiotic use ≤ 3 months before study inclusion, CT related knowledge (infection, transmission routes), attitude questions on CT screening for women and partners (how did they experience the test offer in terms of feeling satisfied, surprised, stigmatized or ashamed), and sexual risk behaviour including age of sexual debut, history of STI, having a new partner in the last 3 months, number of partners in the last 12 months, sex in exchange for money, and condom use. Partners filled in a questionnaire with the same questions on sociodemographic characteristics, lifestyle factors (except alcohol use), antibiotic use, CT related knowledge and attitude questions, sexual risk behaviour, complaints, and history of STI.

Migration background was measured as country of birth of both the woman and her partner and both their parents, and subsequently recoded in Western or non-Western (1st and 2nd generation) migration background. A Western background included Western Europe, North America, Australia and New Zealand. All other regions were defined as non-Western background. Data on socioeconomic status (SES) and urbanity for postal code areas were added to the database. SES status scores were obtained from the Netherlands Institute for Social Research (available at: www.scp.nl). This score takes into account the average income per household in a given postal code area as well as the percentage of households with low income, without paid work, and with low education level. The level of urbanity by postal code area was obtained from Statistics Netherlands (www.cbs.nl).

Women provided vaginal swabs and men provided urine specimens. CT, NG and TV DNA was detected in a two-step approach: first human and microbial DNA was isolated using the High Pure PCR Template Preparation Kit (Roche), and secondly the isolated DNA was used in a PCR system based on the CE-IVD certified PRESTO CT-NG test and the CE-IVD PRESTO TV kit (Goffin Molecular Diagnostics, Beesd, the Netherlands). The assays were performed following the instructions for use of the manufacturer. PCR Cp value’s of 40 and higher were considered negative. The Isolation and Amplification Control (IAC) needs to be positive to consider the sample PCR result reliable. In case the sample is positive for CT, NG or TV the IAC might become negative due to competition, in that case the result is reliable.

Test results were provided by mail in closed envelopes and separately for women and partners, leaving the decision to share test results open. Midwives received the test results for their female clients only. Women and partners who tested positive for CT, NG or TV received an information brochure on the diagnosed STI and a referral letter for their general practitioner (GP) to get treatment.

After birth, pregnancy and neonatal outcomes were collected through pregnancy cards obtained from midwives that included gynaecological information, weeks of gestation, birth weight of the newborn, congenital defects, single or multiple birth(s), medication, preeclampsia, drug use, herpes simplex virus (HSV) infections among women or partners, and STI history of the mother.

### Data analysis

To evaluate the representativeness of the participating midwifery practices, the demographic characteristics of pregnant women from the participating practices were compared to non-participating practices while using aggregated data from Perined (the Dutch national perinatal registry) for the same period. This dataset includes characteristics of pregnant women such as age, ethnicity, weeks of gestation, single or multiple birth(s), and birth weight of the newborn. Descriptive statistics with Chi-square tests were used to compare demographic and obstetric characteristics between (sub)groups.

Knowledge scores were calculated based on twelve questions about CT infection and transmission risks that could be answered with “true”, “false”, or “I don’t know”. Scores were calculated by giving each correct answer a value of 1, and an incorrect answer or ‘I don’t know’ a value of 0. The knowledge sum score could vary between 0 and 12. The knowledge score was included as covariate in the multivariable models in case statistically significant univariably. Exploratory factor analyses were used for the 5-item CT attitude scales to obtain theoretical constructs (factors) for ‘Attitude towards CT screening’ for inclusion as covariate in the multivariable models if relevant. Factors with a Cronbach’s alpha ≥ 0.7 were chosen. For women, the 6 attitude questions resulted in 2 attitude factors of which only one factor (‘negative emotions with the test offer’) was reliable with a Cronbach’s alpha of 0.74 and included two 5-scale (strongly agree to disagree entirely) questions (“I felt ‘stigmatized’ when I was asked to take the Chlamydia test”, “I was embarrassed when I was asked to take the Chlamydia test”). For partners, the same factor was obtained with a Cronbach’s alpha of 0.72.

Univariable and multivariable logistic regression analyses were used to explore risk factors for STI during pregnancy, with the combined STI endpoint of having any of the CT-, NG-, and/or TV-infection(s), due to small numbers of infections. Analyses were repeated for a single CT infection as endpoint, as this was the most common STI and the focus of the study. Multivariable models were adjusted for all covariates as listed with p < 0.20 (Wald test) in univariable analyses using backwards stepwise selection (as listed in footnotes of Tables 2, 3, 4). Highly correlated variables (Spearman correlation coefficients) were not included in the model together; the strongest associated variables were selected or variables were combined into new variables (e.g. combined female and male variables for smoking). Variables were considered statistically significant when p < 0.05 for the likelihood ratio test.

Procedures were repeated for the dichotomous outcome variables of any adverse perinatal outcome (APO) and separately for small-for-gestational-age (SGA). APO was defined as ‘Yes’ if one or more of the variables of preterm birth (< 37 weeks), low birthweight (< 2500 g), premature rupture of membranes (PROM), stillbirth or neonatal conjunctival and respiratory infections were reported. The outcome SGA (birthweight for gestational age < 10th percentile) was calculated according to the Dutch standards [40]. Independent variables of pregnant women and partners included age, migration background, SES, urbanity, religion, education, (history of) STI, drug use, smoking, and antibiotic use. Independent variables for women only included marital status, duration of relationship, body mass index (BMI kg/m^2^), alcohol use, gravity, parity, gestational age at discovery of pregnancy, and gestational age at study inclusion. By including this last variable in the model, we could examine a potential effect of (treated) STI on perinatal outcomes. As previous pregnancies may influence subsequent pregnancy outcomes, sub-analyses of nulliparous women were performed to examine associations between STI and APO in more detail. Analyses were done in SPSS 24.0 (SPSS inc, Chicago, IL).

## Results

### Midwifery practices

The characteristics of pregnant women from the participating practices and non-participating practices were very similar. The percentage of pregnant women of Dutch origin was 72.3% and 73.2% respectively. A small but significant difference was observed for low SES; 39.8% percent versus 38.2% respectively (P < 0.05). Obstetric characteristics including gestational age, prematurity, and neonatal birthweight did not differ significantly between the two groups (Additional file 1: Table S1).

### Demographic characteristics

In total, 856 pregnant women and 575 male partners were registered as being approached for study participation of whom 83 women and 19 partners returned a non-participation form. Reasons for non-participation among women were: not interested (32.5%), recently tested for STI (25.3%), ‘not at risk’ (13.2%), too much on their mind (7.2%), first partner (6.0%), not willing to take a sample (3.6%), and other/unknown reasons (12.0%). Reasons for non-participation among males were often the same as for females (a ‘couple decision’) and were therefore not presented.

All 773 women (90.3%) gave informed consent of whom 548 returned the questionnaire and a vaginal swab (70.9%). These women and their 425 partners (73.9%) were included in the analyses. For 457 women (83.4%), perinatal outcome data from pregnancy cards were available (Table 1). No multiple births were reported.

**Table 1:** Characteristics of pregnant women and partners

	Pregnant women (n/%)	Partners (n/%)	Pregnant women with perinatal outcome data (n/%)
	N = 548	N = 425	N = 457
Demographics*			
Age			
Median (years, IR)	27 (25–29)	29 (27–32)	27 (24–29)
≤ 20	32 (5.8)	9 (2.1)	26 (5.7)
21–25	156 (28.8)	58 (13.6)	138 (30.2)
26–30	360 (65.7)	199 (46.8)	293 (64.1)
≥ 31	0 (0)	157 (36.9)	0 (0)
Age difference (years) with partner			
0–4	392 (71.5)	NA	326 (71.3)
5–9	96 (17.5)		84 (18.4)
≥ 10	33 (6.0)		27 (5.9)
Migration background			
Western	412 (75.2)	333 (78.4)	345 (75.5)
Non-Western	119 (21.7)	78 (18.4)	100 (21.9)
Highest level education			
High	272 (49.6)	192 (45.2)	222 (48.6)
Middle	225 (41.1)	181 (42.6)	196 (42.9)
Low	34 (6.2)	37 (8.7)	27 (5.9)
Socioeconomic status (SES)			
Very high and high SES	117 (21.4)	NA	95 (20.8)
Average SES	211 (38.5)		183 (40.0)
Low and very low SES	197 (35.9)		164 (35.9)
Urbanity			
Very high urbanity	103 (18.8)	NA	83 (18.4)
High urbanity	252 (46.0)		215 (47.0)
Average to low urbanity	173 (31.6)		146 (31.9)
Province			
Noord-Holland	198 (36.1)	NA	173 (37.9)
Zuid-Holland	120 (21.9)		89 (19.5)
Other	210 (38.3)		183 (40.0)
Marital status			
Married or living together	460 (83.9)	NA	388 (84.9)
Single or partner not living together	70 (12.8)		56 (12.3)
Duration relationship (years)			
0–4	193 (35.2)	NA	162 (35.4)
5–9	221 (40.3)		185 (40.5)
≥ 10	84 (15.3)		70 (15.3)
Religion			
No	330 (60.2)	278 (65.4)	276 (60.4)
Yes, Catholic/Christian	169 (30.8)	104 (24.5)	142 (31.1)
Yes, Islamic	27 (4.9)	15 (3.5)	23 (5.0)
Yes, else	1 (0.2)	6 (1.4)	1 (0.2)
Partner (distant) relative			
No	501 (91.4)	NA	420 (91.9)
Yes	4 (0.7)		4 (0.9)
Migration background partner			
Western	394 (71.9)	NA	331 (72.4)
Non-Western	114 (20.8)		95 (20.8)
Highest level education partner			
High	230 (42.0)	NA	185 (40.0)
Low/middle	280 (51.1)		242 (53.0)
*Pregnancy information*			
Pregnancy planned and desired			
Planned and desired	385 (70.3)	NA	327 (71.6)
Not planned, but desired	145 (26.5)		118 (25.8)
Gestational age at discovery (weeks)			
0–4	243 (44.3)	NA	196 (42.9)
5–9	266 (48.5)		233 (51.0)
≥ 10	17 (3.1)		12 (2.6)
Gestational age at study inclusion			
1st trimester (1–12 weeks)	168 (30.7)	NA	143 (31.3)
2nd trimester (13–27 weeks)	242 (44.2)		201 (44.0)
3rd trimester (28–41 weeks)	115 (21.0)		97 (21.2)
BMI (pre-pregnancy, kg/m^2^)			
< 18.5	30 (5.5)	NA	25 (5.5)
18.5–25	354 (64.6)		292 (63.9)
25–30	94 (17.2)		83 (18.2)
≥ 30	40 (7.3)		34 (7.4)
Antibiotics < 3 months			
Yes	45 (8.2)	27 (6.4)	35 (7.7)
No	475 (86.7)	371 (87.3)	401 (87.7)
Gravity			
0	302 (55.1)	NA	242 (53.0)
1	154 (28.1)		136 (29.8)
2 or more	73 (13.3)		65 (14.2)
Parity			
0	362 (66.1)	NA	296 (64.8)
1	130 (23.7)		114 (24.9)
2 or more	20 (3.6)		18 (3.9)
Miscarriage			
No	425 (77.6)	NA	351 (76.8)
Yes	81 (14.8)		71 (15.5)
Abortion			
No	172 (31.4)	NA	151 (33.0)
Yes	50 (9.1)		46 (10.1)
Complication(s) previous newborn		NA	
No	531 (96.9)		442 (96.7)
Yes	17 (3.1)		15 (3.3)
*Behavioural risk factors*			
Age at sexual debut (years)			
< 18	354 (64.6)	215 (50.6)	301 (65.9)
≥ 18	146 (26.6)	151 (35.5)	120 (26.3)
No. of sex partners in last 12 months			
0–1 partner	492 (89.8)	377 (88.7)	415 (90.8)
2 or more	24 (4.4)	13 (3.1)	19 (4.2)
New sex partner in last 3 months			
No	521 (95.1)	398 (93.6)	438 (95.8)
Yes	7 (1.3)	2 (0.5)	6 (1.3)
Condom use at start current relation			
Always/mostly to regular	326 (59.5)	246 (57.9)	274 (60.0)
Not always to never	191 (34.9)	147 (34.6)	159 (34.8)
STI-related symptoms			
0	217 (39.6)	374 (88.0)	180 (39.4)
1	171 (31.2)	24 (5.6)	145 (31.7)
2	100 (18.2)	2 (0.5)	87 (19.0)
3 or more	42 (7.7)	0 (0.0)	33 (7.2)
Previous STI test			
No	237 (43.2)	248 (58.4)	202 (44.2)
Yes	289 (52.7)	149 (35.1)	238 (52.1)
History of STI	83 (28.7)	45 (30.2)	71 (29.8)
No history of STI	198 (68.5)	101 (67.8)	161 (67.6)
Smoking			
No, never	202 (36.9)	124 (29.2)	173 (37.9)
In the past, not during pregnancy	208 (38.0)	169 (39.8)	169 (37.0)
Yes, during pregnancy	120 (21.9)	108 (25.4)	102 (22.3)
Yes, stopped since pregnant	77 (14.1)	13 (3.1)	65 (14.2)
Yes, during pregnancy	43 (7.8)	95 (22.4)	37 (8.1)
Alcohol			
No, never	51 (9.3)	NA	41 (9.0)
In the past, not during pregnancy	111 (20.3)		96 (21.0)
Yes, during pregnancy	365 (66.6)		305 (69.0)
Yes, stopped since pregnant	354 (64.6)		294 (64.4)
Yes, continued since pregnancy	11 (2.0)		11 (2.4)
Drugs			
No, never	343 (62.6)	217 (51.1)	293 (64.1)
In the past, not during pregnancy	162 (29.6)	134 (31.5)	132 (28.9)
Soft drugs only	111 (68.5)	81 (60.4)	90 (68.2)
Hard drugs ± soft drugs	49 (30.2)	51 (38.1)	40 (30.3)
Yes, during pregnancy	23 (4.2)	47 (11.1)	17 (3.7)
Soft drugs only	17 (73.9)	22 (55.0)	13 (76.5)
Hard drugs (or both)	6 (26.1)	18 (45.0)	4 (23.5)
Yes, stopped since pregnant	22 (4.0)	7 (1.6)	17 (3.7)
Yes, continued since pregnant	1 (0.2)	40 (9.4)	0 (0.0)
STIs (95% CI)	13 (2.4, 1.3–4.0)	10 (2.2, 1.0–4.0)	11 (2.4, 1.2–4.3)
Chlamydia	10 (1.8, 0.9–3.3)	10 (2.2, 1.0–4.0)	9 (2.0, 0.9–3.7)
Gonorrhoea	2 (0.4, 0.0–1.3)	1 (0.2, 0.0–1.3)	1 (0.2, 0.0–1.2)
Trichomoniasis	2 (0.4, 0.0–1.3)	0 (0.0, 0.0–0.9)	1 (0.2, 0.0–1.2)
More than 1 STI	1 (0.2, 0.0–1.2)	1 (0.2, 0.0–1.3)	0 (0.0, 0.0–0.9)
CT knowledge			
CT knowledge score (median, IQR)	9.0 (8.0–11.0)	9.0 (7.0–10.0)	9.0 (7.0–11.0)
0–5 points (low score)	59 (10.8)	65 (15.3)	54 (11.8)
6–9 points (middle score)	221 (40.3)	184 (43.3)	181 (39.6)
10–12 points (high score)	249 (45.4)	153 (36.0)	209 (45.7)
*Pregnancy outcomes*			
Adverse perinatal outcome			
No	397 (72.4)		397 (86.9)
Yes	50 (9.1)		50 (10.9)
Prematurity (< 37 weeks)			
No	417 (76.1)		417 (91.2)
Yes	32 (5.8)		32 (7.0)
Premature rupture of membranes			
No	433 (79.0)		433 (94.7)
Yes	18 (3.3)		18 (3.9)
Low birthweight (< 2500 g)			
No	426 (77.7)		426 (93.2)
Yes	19 (3.5)		19 (4.2)
Small for gestational age			
No	418 (76.3)		418 (91.5)
Yes	28 (5.1)		28 (6.1)

*NA* not applicable or not asked

^*^Totals vary due to missing values. Information missing for 0.7 to 8.8% for questionnaire variables (exception: information on abortion was missing for 56%). Information missing for 0 to 7.7% for perinatal outcomes from pregnancy cards

The median age at enrolment for women was 27 years [interquartile range (IR): 25–29 years] and 29 years [IR: 27–32] for their partners. Most participants had a Dutch or other Western background (75.2% of women, 78.4% of men), and half of the participants had a high-level education (49.6% of women and 53.4% of men). Most women were married or living together but unmarried (83.9%). Thirty-six percent of the women had a religion of whom the majority was Catholic or had another Christian religion, and 4.9% were Muslim.

### Lifestyle factors

During pregnancy 21.9% of the women smoked of whom 64.2% stopped after the pregnancy became known and 7.8% smoked during the entire pregnancy (Table 1). Of their partners, 25.4% smoked during the pregnancy, but only 12.0% stopped when the pregnancy was known. Alcohol use by women was 67%, but 97% stopped when the pregnancy was discovered. Any drug use among women was 4.2% of whom 73.9% used soft drugs and 26.1% used hard drugs. One woman (0.2%) continued to use soft drugs during pregnancy; none were using hard drugs. Of the partners, 11.1% used drugs during the pregnancy and 9.4% continued to use drugs during pregnancy (55.0% soft drugs, 45.0% hard drugs or combination of both).

### STI positivity during pregnancy

The pooled STI positivity (CT, NG and TV) was 2.4% (95%CI 1.3–4.0%) for the women and 2.2% (CI 1.0–4.0) for their partners. Among the women, 1.8% (CI 0.9–3.3) was infected with CT, 0.4% (CI 0.0–1.3) with NG and 0.4% (CI 0.0–1.3) with TV (Table 1); among men CT positivity was 2.2% (CI 1.0–4.0), NG positivity 0.2% (CI 0.0–1.3), and no TV infections were identified. CT positivity was 12.5% (CI 3.5–29.0) among women aged ≤ 20 years of age, 3.1% (CI 0.8–7.6) among women < 25 years, and 1.4% (CI 0.3–3.5) among women of 20–25 years of age.

Discordant CT test results within couples were as follows: 14% of the male partners were CT negative while the female was CT positive, and among CT positive male partners, 40% of the women were CT negative.

Women had slightly more knowledge on CT infection and transmission risks than the partners: 13.4% of the women answered all questions correct compared to 10.4% of the men (Table 1).

Univariable logistic regression analyses showed that an STI diagnosis during pregnancy was associated with various demographic, behavioural, and lifestyle variables (Table 2). Adjusted for covariates, three variables remained in the model: young age at inclusion (≤ 20 years versus ≥ 21 years, adjusted OR (aOR) 6.62, CI 95%: 1.11–38.33), having a partner of non-Western background vs Western background (aOR 9.34, CI 2.34–37.21), and having two or more sex partners in the past 12 months (aOR 9.88, CI 2.08–46.91).

**Table 2:** Risk factors of women and partners associated with STI during pregnancy

Factor	Crude OR (95% CI)	p-value	Adjusted OR^a^ (95% CI)	p-value
Age group: ref = ≥ 21 years	1		1	
≤ 20 years	**8.05 (2.33–27.75)**	**0.001**	**6.52 (1.11–38.33)**	**0.04**
Migration background: ref = Western	1			
Non-Western	**5.09 (1.58–16.33)**	**0.006**		
Migration background partner: ref = Western	1			
Non-Western	**9.84 (2.56—37.72)**	**0.001**	**9.34 (2.34–37.21)**	**0.002**
Education: ref = high	1			
Low/middle^b^	3.23 (0.86–12.06)	0.08		
Education partner: ref = high	1			
Low/middle^b^	3.79 (0.81–17.70)	0.09		
Marital status: ref = married/living together	1			
Single or partner not living together	**4.98 (1.54–16.15)**	**0.008**		
Duration relation: ref = ≤ 5 years	1			
0–4 years	3.79 (0.97–14.83)	0.06		
Age at sexual debut: ref = 18 +	1			
< 18	4.65 (0.60–36.35)	0.14		
New sex partner < 3 months: ref = no	1			
Yes	7.73 (0.86–69.71)	0.07		
Nr sex partners < 12 months: ref = 0–1	1			
Two or more	**12.10 (3.36–43.56)**	**0.0001**	**9.88 (2.08–46.91)**	**0.004**
Vaginal discharge: ref = no	1			
Yes	0.32 (0.07–1.49)	0.15		
Pain or burning sensation when urinating: ref = no	1			
Yes	3.23 (0.85–12.33)	0.09		
History of STI: ref = not tested or no STI	1			
1 or more STI	**7.05 (2.31–21.53)**	**0.001**		
Partner history of STI: ref = not tested or no STI	1			
1 or more STI	3.35 (0.89–12.61)	0.07		
Smoking: ref = no never or not during pregnancy	1			
Yes during pregnancy	**3.54 (1.12–11.20)**	**0.03**		
Partner smoking: ref = no never or not during pregnancy	1			
Yes during pregnancy	3.54 (0.93–13.45)	0.06		
Drug use: ref = no never or not during pregnancy	1			
Yes during pregnancy	4.71 (0.97–22.88)	0.05		
Partner using drugs: ref = no never or not during pregnancy	1			
Yes during pregnancy	**7.09 (1.53–32.92)**	**0.01**		
Pregnancy planned: ref = yes	1			
No	**3.85 (1.20–12.35)**	**0.02**		
Antibiotic < 3 months: ref = no	1			
Yes	**5.70 (1.65–19.72)**	**0.006**		

Non-significant variables or not available/applicable (NA) results are not shown: calendar year, age partner, age difference, SES, urbanity, province, gestational age in trimesters at study inclusion, gestational age at discovery of pregnancy, female religion, partner religion, antibiotic use < 3 months partner, partner complaints, duration of becoming pregnant in years, previous pregnancies, abortion(s), miscarriage(s), complications previous newborn, parity, gravity, vaginal bleeding, blood loss during or after sex, pain in lower abdomen, condom use, partner with new sex partner < 3 months, male number of sex partners in past 12 months, age of sexual debut partner, female alcohol use, female CT knowledge score, partner CT knowledge score, negative emotions with the test offer (female), test offer had no negative impact on me (female), STI diagnosis of the partner (highly correlated variable, but not included due to small numbers and inflated crude OR value)

Multivariable model adjusted for: female age, female migration background, female education, number of partners < 12 months, history of STI, female smoking, antibiotic use

Significant associations are shown in bold

^a^adjusted by backward stepwise method; OR odds ratios; CI confidence interval; STI sexually transmitted infection(s)

^b^Categories combined due to small numbers

The same model was run for CT infection as outcome variable. Results were the same, but confidence intervals were wider due to smaller numbers: age ≤ 20 years at inclusion (aOR 11.45, CI 1.80–72.90), having a non-Western partner (aOR 9.45, CI 1.84–49.50) and having two or more sex partners in the past 12 months (aOR 9.62, CI 1.54–60.25) (model not shown).

### Adverse perinatal outcomes

Of the pregnant women 10.9% had one or more adverse perinatal outcome(s) (APO), including preterm birth (< 37 weeks, 7.0%), premature rupture of membranes (PROM, 3.9%), low birthweight (< 2500 g, 4.2%), stillbirth (n = 1, 0.22%), and neonatal conjunctival infection (n = 1, 0.22%). No neonatal respiratory infections were reported.

Having an STI during pregnancy, having had a history of STI diagnoses, and/or having a partner with a current STI diagnosis were not associated with APO (Table 3). CT infection during pregnancy was also not associated with APO (not shown).

**Table 3:** Factors associated with adverse perinatal outcomes (APO)

Factor	Crude OR (95% CI)	p-value	Adjusted OR^a^ (95% CI)	p-value
Migration background: ref = Western	1		1	
Non-Western	1.51 (0.78–2.95)	0.22		
Education: ref = high	1		1	
Middle	1.72 (0.89–3.30)	0.1	1.44 (0.71–2.89)	0.31
Low	**4.16 (1.54–11.22)**	**0.005**	**3.36 (1.12–10.09)**	**0.03**
Marital status: ref = married/living together	1			
Single or not living with partner	2.11 (0.98–4.53)	0.06		
Duration relation: ref = ≥ 5 years	1		1	
0–4 years	**2.82 (1.50–5.30)**	**0.001**	**2.75 (1.41–5.39)**	**0.003**
Pain in lower abdomen: ref = no	1			
Yes	1.72 (0.94–3.15)	0.08		
Blood loss during or after sex: ref = no	1			
Yes	0.24 (0.03–1.81)	0.17		
Antibiotic < 3 months: ref = no	1			
Yes	0.23 (0.03–1.71)	0.15		
BMI: ref = 18.5–25	1			
< 18.5	2.33 (0.81–6.71)	0.12		
25–30	0.83 (0.35–1.96)	0.67		
≥ 30	1.53 (0.55–4.25)	0.42		
Complications previous newborn: ref = no	1		1	
Yes, 1 or more	**7.92 (2.74–22.90)**	**0.001**	**10.49 (3.21–34.25)**	**0.0001**
Nr sex partners < 12 months: ref = 0–1	1			
2 or more	2.32 (0.74–7.31)	0.15		
Current STI: ref = no	1			
Yes, 1 or more	0.88 (0.11–7.09)	0.9		
History of STI: ref = no	1			
Yes, 1 or more	1.46 (0.69–3.08)	0.32		

Non-significant (ns) variables or not available/applicable (NA) results are not shown: calendar year, female age, age partner, age difference, SES, urbanity, province, migration background partner, female religion, religion partner, education partner, pre-eclampsia (n = 18, 3.9%), female HSV infection (n = 4, 0.9%), male HSV infection (n = 18, 5.1%), gravity, parity, gestational age in trimesters at study inclusion, gestational age at discovery of pregnancy, planned pregnancy, miscarriage(s), abortion(s), female drug use, partner drug use, female smoking, partner smoking, female alcohol use, vaginal bleeding, pain or burning sensation when urinating (female), vaginal discharge, condom use in current relation, number of sex partners < 12 months (female), new sex partner < 3 months (female), partner is (distant) family, number of sex partners < 12 months (male), new sex partner < 3 months (male), age at sexual debut (female), partner having current STI, partner history of STI, partner antibiotic use < 3 months, female CT knowledge score, partner CT knowledge score

Multivariable model adjusted for: female education, duration current relationship, complications previous newborn, female antibiotic use < 3 months, BMI

Significant associations are shown in bold

^a^Adjusted by backward stepwise method; OR odds ratios; CI confidence interval; STI sexually transmitted infection(s)

However, other factors that were associated with APO were complications with the previous newborn (preterm birth and/or low birthweight) (aOR 10.49, CI 3.21–34.25 vs no complications), short duration of the relationship (aOR for 0–4 years: 2.75, CI 1.41–5.39 vs ≥ 5 years) and female low education (aOR 3.36, CI 1.12–10.09).

As a substantial proportion of the pregnant women had previous deliveries (26.6%) that may influence subsequent pregnancy outcomes, the same analyses were done for nulliparous women. The APO model for nulliparous women showed that none of the STI-variables were associated with APO. However, short duration of the relationship was still associated with APO (aOR for 0–4 years: 3.52, CI 1.47–8.43 vs ≥ 5 years), and an additional risk factor was obesity (BMI ≥ 30, aOR 4.46, CI 1.21–16.47 vs BMI 18.5–25, Additional file 1: Table S2).

Of the newborns, 28 (6.3%) were small for gestational age (SGA). The univariable models for SGA showed that female and male non-Western migration background, female low education level, province, female and male smoking, and having had an STI in the past (female) were associated with SGA (Table 4). Having an STI (or CT only) in the current pregnancy was not associated with SGA univariably and was not included in the adjusted model. Factors that remained associated in the adjusted model were: female low education (aOR 7.81, CI 2.01–30.27), female non-Western background (aOR 4.41, CI 1.74–11.17), and both female and male smoking during pregnancy (aOR 2.94, CI 1.01–8.84).

**Table 4:** Factors associated with small for gestational age newborns

Factor	Crude OR (95% CI)	p-value	Adjusted OR^a^ (95% CI)	p-value
Migration background women: ref = Western	1		1	
Non-Western	**3.64 (1.65–8.03)**	**0.01**	**4.41 (1.74–11.17)**	**0.002**
Migration background partner: ref = Western	1			
Non-Western	**2.55 (1.11–5.89)**	**0.03**		
Education women: ref = high	1		1	
Middle	1.54 (0.64–3.74)	0.34	1.36 (0.50–3.71)	0.55
Low	**6.93 (2.24–21.47)**	**0.001**	**7.81 (2.01–30.27)**	**0.003**
Education partner: ref = high	1			
Middle	1.46 (0.58–3.65)	0.42		
Low	2.96 (0.92–9.55)	0.07		
Marital status: ref = married/living together	1			
Single or not living with partner	2.14 (0.82–5.56)	0.12		
Duration relation: ref = ≥ 5 years	1			
0–4 years	2.13 (0.94–4.81)	0.07		
Province: ref = Noord-Holland	1			
Zuid-Holland	**0.19 (0.04–0.84)**	**0.03**		
Else	**0.37 (0.16–0.89)**	**0.03**		
Vaginal discharge: ref = no	1			
Yes	0.37 (0.14–1.00)	0.05		
Smoking^b^: ref = both never or not in pregnancy	1		1	
Woman or partner in pregnancy	1.92 (0.67–5.50)	0.22	1.56 (0.51–4.81)	0.44
Woman and partner in pregnancy	**3.54 (1.27–9.84)**	**0.02**	**2.94 (1.01–8.84)**	**0.05**
Drug use: ref = no never or not during pregnancy	1			
Yes during pregnancy	3.49 (0.94–12.98)	0.06		
Gestational age at inclusion: ref = first trimester	1			
Second trimester	0.40 (0.16–1.00)	0.05		
Third trimester	0.63 (0.23–1.72)	0.36		
Gestational age at discovery pregnancy: ref = 0–5 weeks	1			
5–10 weeks	1.89 (0.80–4.48)	0.15		
≥ 10 weeks	5.17 (0.96–27.94)	0.06		
Pregnancy planned: ref = yes	1			
No	1.76 (0.78–3.98)	0.17		
Current STI, ref = no	1			
Yes, 1 or more	1.68 (0.21–13.78)	0.63		
History of STI: ref = no	1			
Yes, 1 or more	**2.62 (1.14–6.05)**	**0.02**		

Significant associations are shown in bold

^a^Adjusted by backward stepwise method; OR odds ratios; CI confidence interval; STI sexually transmitted infection(s). Non-significant (ns) variables or not available/applicable (NA) results are not shown: calendar year, female age, age partner, age difference, SES, urbanity, female religion, partner religion, pre-eclampsia, female HSV infection, partner HSV infection, parity, gravity, miscarriage(s), abortion(s),, female drug use, partner drug use, female alcohol use, partner history of STI, vaginal bleeding, pain or burning sensation when urinating (female), pain in lower abdomen, blood loss during or after sex, condom use in current relation, number of sex partners < 12 months (female), new sex partner < 3 months (female), partner is (distant) family, new sex partner < 3 months (male), age at sexual debut (female), female antibiotic use < 3 months, antibiotic use partner < 3 months, complications previous newborn, BMI, female CT knowledge score, partner CT knowledge score

^b^ Univariable: Smoking female OR 2.42 (CI 1.09–5.40), p = 0.03; Smoking male OR 2.55 (CI 1.08–6.00), p = 0.03. Multivariable model adjusted for: female migration background, female education, duration relationship, province, female history of STI, smoking during pregnancy, female drug use, pregnancy planned, stage of discovery pregnancy

## Discussion

The pooled prevalence of STI (CT, NG and TV) was 2.4% among pregnant women ≤ 30 years of age and 2.2% among their male partners in Dutch midwifery practices. Risk factors for STI during pregnancy were female young age (≤ 20 years), male non-Western background, and having had two or more sex partners in the past 12 months (female). Of the women, 10.9% had one or more adverse perinatal outcomes (APO) and 6.3% of the newborns were small for gestational age (SGA). However, having an STI during pregnancy was not associated with APO nor with SGA in our study population. We did identify other risk factors for APO (low female education, previous newborn with preterm birth, short duration of the current relationship, obesity) and for SGA (low female education, female non-Western background, combined female and male smoking during pregnancy).

The strength of our study is the inclusion of samples and epidemiological information of both pregnant women and their male partners. However, our study also has some limitations. First, the study sample of 548 women of whom 457 had perinatal outcome data is rather small. Together with the low prevalence of STI, this may have caused a lack of statistical power to identify STI associated determinants with small effects that are known for these STI and APO [41, 42]. Second, women were included at various stages of pregnancy. Most (65.1%) women were included in the second or third trimester, which may have attenuated underlying associations with STI due to foetal development and treatment. Prior studies showed that associations between STI during pregnancy and APO are strongest during the first trimester [42]; the stage in which vital organs develop. Third, our study may not be fully representative for all pregnant women in the Netherlands, although we assume no major selection bias in midwifery practices since the baseline characteristics of pregnant women and perinatal outcomes were comparable between participating and non-participating practices. However, our study sample only includes women who understand Dutch. Therefore, percentages of STI as well as APO may have been underestimated, assuming that having a non-Western background is associated with a higher STI prevalence [43, 44]. Finally, we cannot rule out response bias from women and partners due to stigma or social desirability, which could understate exposure to sexual risks or other lifestyle factors during pregnancy.

Previous research, including meta-analyses, showed small but increased odds of APO due to infections with CT and NG during pregnancy, such as preterm delivery and low birthweight [16, 36, 41, 45]. We could not confirm these associations, probably due to the small study sample and low STI prevalence. Also, we assume that most women who were diagnosed with an STI received treatment that may have mitigated any effect of STI on APO. Unfortunately, follow-up information on treatment by general practitioners was not collected. However, we identified other risk factors than STI for APO and SGA newborns, such as low female education. These associations confirm previous findings [46]. Women with low education might be less capable to adopt healthy behaviours before conception or during pregnancy [13, 14, 47]. Education on healthy behaviour before conception and during pregnancy is relevant to reduce APO, especially for women with obesity, which was also associated with APO among nulliparous women [48]. Further, having a previous newborn with low birthweight or preterm birth was a risk factor for APO, confirming earlier studies showing that previous preterm birth was a risk factor for current preterm birth and premature rupture of membranes [49, 50]. Of the lifestyle factors, a small effect of combined male and female smoking was identified for SGA newborns, but other lifestyle factors were not significant. Likely, most women stop smoking or drinking alcohol after finding out about the pregnancy [51, 52]. The role of relationship duration in APO could be the result of underlying factors. A short duration of the relationship could be associated to changing sexual partners that increases the risk of STI or could be related to other risk factors for APO that we could not identify.

The CT prevalence among pregnant women in our study was 1.8% and among women ≤ 20 years of age it was 12.5%. Young age is one of the most important risk factors for CT among women [44, 53]. The Rotterdam study among pregnant women showed an overall CT prevalence of 3.9% (including women up to ≥ 30 years of age), and a prevalence of 13.5% among women ≤ 20 years. Besides young age, Antillean ethnicity, and not being married were independent risk factors for CT infection during pregnancy in the Rotterdam study [36]. In our study, having a non-Western partner and having two or more sex partners in the past 12 months were risk factors for CT infection. The partners’ non-Western background was stronger associated than the female non-Western background, although the two are interrelated [54]. This relationship between migration background of the partner and CT infections among women has been previously described [44, 53]. In our study, the group of people with a non-Western background also included men and women with an Antillean or Surinamese background. In the Rotterdam study, CT prevalences among Antillean women and Surinamese women were 16.2%, and 9.1% respectively. Prevalence rates among these subgroups in our study were not shown due to small numbers. Therefore, we used the variable Western and non-Western background. Small numbers could also be the reason that female migration background was not significant in the regression models.

We also showed that male partners can be a critical source of transmission to pregnant women who tested negative for CT. Among CT infected male partners, 40% of the women tested CT negative. These women are at risk of transmission during their pregnancy or in the post-delivery phase. Although this proportion may not accurately reflect discordant rates due to small numbers, male involvement in CT testing has been recognized internationally as an important aspect of prevention of mother-to-child transmission [7, 55]. In general, CT testing during pregnancy is well accepted by women and their partners [39].

International guidelines recommend antenatal screening for CT of all women under 25 years of age and of older women at increased (sexual) risk [7]. These guidelines are not followed in the Netherlands. A previous study showed that Dutch midwives base their decisions to offer CT testing to pregnant women on symptoms rather than on risk profiles [38]. Hence, since up to 80% of CT infections in women and 50% in men remain asymptomatic [7], most pregnant women will remain undetected with the current prenatal Dutch standard. Subsequently, CT infected women are left untreated with a risk for postpartum PID for themselves, a risk for transmission to their offspring with possible neonatal conjunctivitis and pneumonia, and a risk for transmission to their partner. The high CT prevalence (12.5%) we found among women ≤ 20 years in this study coincides with the high 13.5% prevalence in the same age group in the Rotterdam study. These findings would support the implementation of the international guidelines. Also, CT screening of this group of young pregnant women (≤ 20 years) was shown to be particularly cost-saving based on the results from the Rotterdam study [36, 37]. Based on this study, screening of women age < 25 years would also be cost-effective; the prevalence in this age group was 3.1% compared to 6.7% in the Rotterdam study. However, to decide on a national CT screening program for all pregnant women in the Netherlands, more research might be needed on its (cost)-effectiveness [37, 56–58].

In conclusion, the prevalence of STI among pregnant women, and their male partners, in Dutch midwifery practices was low, except for a high prevalence of CT infection among young women. Important female and male risk factors for STI and for CT infection during pregnancy were identified as young age, having had two or more sex partners in the past 12 months, and having a male partner with a non-Western background. Previously observed associations between STI and APO could not be confirmed. In the absence of a national screening program midwives and obstetricians should be aware of the high prevalence of CT among women under the age of 20 and with multiple sexual partners.

## Supplementary Information

**Additional file 1: Table S1.** Characteristics of pregnant women at participating and non-participating midwifery practices in the Netherlands (Source: Perined). **Table S2.** Factors associated with adverse perinatal outcomes (APO), nulliparous women.

## Data Availability

The dataset presented in this article is not publicly available because statistical information or data for research purposes can be requested by submitting a research proposal. Requests to access the dataset should be directed to the corresponding author.

## Abbreviations

STI: Sexually transmitted infection
CT: *Chlamydia trachomatis*
NG: *Neisseria gonorrhoeae*
TV: *Trichomonas vaginalis*
HIV: Human immunodeficiency virus
HBV: Hepatitis B virus
HSV: Herpes simplex virus
APO: Adverse perinatal outcomes
SGA: Small for gestational age
PROM: Premature rupture of membranes
PID: Pelvic inflammatory disease
BMI: Body mass index
IC: Informed consent
GP: General practitioner
SES: Socio economic status
WHO: World Health Organization
OR: Odds ratio
CI: Confidence interval
DNA: Deoxyribonucleic acid
PCR: Polymerase chain reaction
IAC: Isolation and amplification control
CE-IVD: European Community marking for in vitro diagnostic devices
