# Supplementary material for: Chlamydia trachomatis, Neisseria gonorrhoea, and Trichomonas vaginalis infections among pregnant women and male partners in Dutch midwifery practices: prevalence, risk factors, and perinatal outcomes
Source: Reprod Health. 2021 Jun 26;18:132. doi: 10.1186/s12978-021-01179-8 (PMC8236142; doi:10.1186/s12978-021-01179-8)
Supplement: Supplementary file 1 — Additional file 1: Table S1. Characteristics of pregnant women at participating and non-participating midwifery practices in the Netherlands (Source: Perined). Table S2. Factors associated with adverse perinatal outcomes (APO), nulliparous women. [file 12978_2021_1179_MOESM1_ESM.docx]

**Supplementary table**

*Representativeness midwifery practices*

To examine the representativeness of the participating midwifery practices (covering approximately 5.0% of pregnant women in the Netherlands), we compared both the demographic baseline characteristics of pregnant women and the obstetric data from participating and non-participating midwifery practices in the Netherlands from the national perinatal registry (Perined, Table 1). Ethnicity of the pregnant women did not differ significantly between participating and non-participating practices, but a small difference was observed between the proportions of socioeconomic status. Obstetric data (prematurity, gestational age, and birth weight) did not differ between participating and non-participating practices.

Table 1. Characteristics of pregnant women at participating and non-participating midwifery practices in the Netherlands (Source: Perined).

|  | Participating  practices (n/%) | Non-participating practices  (n/%) | Chi-square,  p-value |
| --- | --- | --- | --- |
| Ethnicity |  |  | p=0.08 |
| - Dutch | 5,911 (72.3%) | 109,815 (73.2%) |  |
| - Mediterranean | 646 (7.9%) | 11,256 (7.5%) |  |
| - Other European | 418 (5.1%) | 7,546 (5.0%) |  |
| - mixed/other | 536 (6.6%) | 10,877 (7.3%) |  |
| Socioeconomic Status |  |  | p<0.05 |
| - very wealthy/wealthy | 1,805 (22.6%) | 35,152 (23.6%) |  |
| - average | 3,002 (37.6%) | 59,881 (40.3%) |  |
| - less-favoured/deprived | 3,173 (39.8%) | 56,820 (38.2%) |  |
| Weeks of Gestation (weeks) |  |  | p=0.17 |
| - 22.0 - < 25.0 | 27 (0.3%) | 437 (0.3%) |  |
| - 25.0 - < 28.0 | 24 (0.3%) | 377 (0.3%) |  |
| - 28.0 - < 31.0 | 41 (0.5%) | 622 (0.4%) |  |
| - 31.0 - < 34.0 | 97 (1.2%) | 1,459 (1.0%) |  |
| - 34.0 - < 37.0 | 343 (4.2%) | 6,932 (4.7%) |  |
| - 37.0 - < 41.0 | 6,142 (75.8%) | 112,703 (75.7%) |  |
| - >= 41.0 | 1,431 (17.7%) | 26,365 (17.7%) |  |
| Prematurity  - no  - yes (<37 weeks)  Birth weight (grams) | 7,573 (93.4%)  532 (6.6%) | 139,068 (93.4%)  9,827 (6.6%) | p=0.89  p=0.17 |
| - < 1500 | 99 (1.2%) | 1,633 (1.1%) |  |
| - 1500 - < 2000 | 93 (1.1%) | 1,550 (1.0%) |  |
| - 2000 - < 2500 | 293 (3.6%) | 5,283 (3.5%) |  |
| - 2500 - < 3000 | 1,272 (15.6%) | 22,216 (14.7%) |  |
| - 3000 - < 3500 | 2,809 (34.4%) | 53,766 (35.7%) |  |
| - 3500 - < 4000 | 2,604 (31.9%) | 48,020 (31.9%) |  |
| - 4000 - < 4500 | 869 (10.6%) | 15,731 (10.4%) |  |
| - >= 4500 gram | 127 (1.6%) | 2,499 (1.7%) |  |

Table 2. Factors associated with adverse perinatal outcomes (APO), nulliparous women

| Factor | Crude OR (95% CI) | p-value | Adjusted OR^a^ (95% CI) | p-value | |
| --- | --- | --- | --- | --- | --- |
| Education: ref=high  - middle  - low | 1  1.54 (0.69-3.42)  **4.65 (1.40-15.43)** | 0.29  **0.01** |  |  | |
| Duration relation: ref=≥ 5 years  - 0-4 years | **1**  **3.17 (1.40-7.18)** | **0.006** | **1**  **3.52 (1.47-8.43)** | **0.005** | |
| BMI: ref=18.5-25  <18.5  25-30  ≥30  Current STI: ref=no  - yes, 1 or more  History of STI: ref=no  - yes, 1 or more | 1  3.20 (0.94-10.92)  1.06 (0.37-3.02)  2.98 (0.88-10.05)  1  1.14 (0.14-9.60)  1  1.74 (0.73-4.14) | 0.06  0.91  0.08  0.90  0.21 | 1  2.74 (0.77-9.68)  1.26 (0.43-3.68)  **4.46 (1.21-16.47)** | | 0.12  0.68  **0.02** |

^a^ adjusted by backward stepwise method; OR odds ratios; CI confidence interval; STI sexually transmitted infection(s).

Non-significant (ns) variables or not available/applicable (NA) results are not shown: calendar year, female age, age partner, age difference, SES, urbanity, province, migration background female, migration background partner, female religion, religion partner, education partner, marital status, pre-eclampsia, female HSV infection, male HSV infection, gravity, gestational age in trimesters at study inclusion, gestational age at discovery of pregnancy, planned pregnancy, duration of getting pregnant, miscarriage(s), abortion(s), female alcohol use, female drug use, partner drug use, female smoking, partner smoking, burning sensation when urinating (female), pain lower abdomen, vaginal discharge, blood loss during or after sex, condom use in current relation, number of sex partners<12 months (female), new sex partner<3 months (female), partner is (distant) family, number of sex partners<12 months (male), new sex partner<3 months (male), age at sexual debut (female), age at sexual debut (male), partner having current STI, partner history of STI, antibiotic use< 3 months (female), partner antibiotic use <3months, female CT knowledge score, partner CT knowledge score

Multivariable model adjusted for: female education, duration current relationship, female antibiotic use<3 months, BMI.
